# Postoperative Complications After Prophylactic Thyroidectomy for Very Young Patients With Multiple Endocrine Neoplasia Type 2: Retrospective Cohort Analysis

**DOI:** 10.1097/MD.0000000000001108

**Published:** 2015-07-24

**Authors:** Wouter P. Kluijfhout, Dirk-Jan van Beek, Annemarie A. Verrijn Stuart, Lutske Lodewijk, Gerlof D. Valk, David C. van der Zee, Menno R. Vriens, Inne H.M. Borel Rinkes

**Affiliations:** From the Department of Surgical Oncology and Endocrine Surgery, University Medical Center Utrecht (WPK, D-JVB, LL, MRV, IHMBR); Department of Pediatric Endocrinology, Wilhelmina Children's Hospital (AAVS); Department of Endocrinology; University Medical Center Utrecht (GDV); and Department of Pediatric Surgery, Wilhelmina Children's Hospital, Utrecht, The Netherlands (DCVDZ).

## Abstract

The aim of this study was to investigate whether younger age at surgery is associated with the increased incidence of postoperative complications after prophylactic thyroidectomy in pediatric patients with multiple endocrine neoplasia (MEN) 2.

The shift toward earlier thyroidectomy has resulted in significantly less medullary thyroid carcinoma (MTC)-related morbidity and mortality. However, very young pediatric patients might have a higher morbidity rate compared with older patients. Hardly any literature exists on complications in the very young.

A retrospective single-center analysis was performed on the outcomes of MEN2 patients undergoing a prophylactic total thyroidectomy at the age of 17 or younger. Forty-one MEN2A and 3 MEN2B patients with thyroidectomy after January 1993 and at least 6 months of follow-up were included, subdivided in 9 patients younger than 3 years, 15 patients 3 to 6 years, and 20 patients older than 6 years. Postoperative hypocalcemia and other complications were registered.

Twelve (27%) patients developed transient hypocalcemia and 9 (20%) patients suffered from permanent hypocalcemia, with a nonsignificant trend toward higher incidence with decreasing age. Three (7%) patients had other complications, of whom 2 were younger than 3 years.

For patients younger than 3 years, the average length of stay (LOS) was 6.7 days, versus 1.7 and 3.5 days, respectively, for the older patient groups (*P* < 0.05). Patients with complications had a longer LOS compared with patients without (5.0 vs 2.0, *P* < 0.01).

None of the patients had clinical signs of recurrent MTC after a mean follow-up of 10.5 years.

Prophylactic thyroidectomy in very young children is associated with a higher rate of complications, causing a significant increased LOS. Irrespective age of surgery, MTC did not recur in any patient. In planning optimal timing of surgery, clinicians should take the risk of complications into account. We advise not to perform total thyroidectomy before the age of 3 for patients defined high risk by the American Thyroid Association guideline.

## INTRODUCTION

Medullary thyroid carcinoma (MTC) arises from calcitonin-producing C-cells in the thyroid gland. Around 25% of the MTC cases are hereditary due to germline mutations on chromosome 10, activating the REarranged during Transfection (RET) proto-oncogene.^1^ These mutations are inherited in an autosomal dominant pattern and cause the endocrine syndromes multiple endocrine neoplasia (MEN) type 2A and MEN type 2B. Before genetic testing became available in 1993, most patients were identified with MTC at a clinical stage causing high morbidity and mortality.^2^ The aggressiveness and age-related penetrance of MTC, however, are strongly related to the genotype.^3^ Nowadays, DNA analysis during early childhood permits early identification of the carriers. Although MEN2A and MEN2B have different clinical manifestations, almost all carriers of mutations specific for these syndromes will develop MTC. Therefore, guidelines were developed to provide mutation-specific recommendations for the optimal timing and extent of surgery, of which the most recent derives from the American Thyroid Association (ATA).^4^ Guideline implementation has caused a shift toward earlier surgery, thereby resulting in less MTC-related morbidity and mortality.^5^ However, in studies regarding pediatric thyroid surgery, complication rates appear to be higher than in adults.^6^ The most common complication after thyroid surgery is hypocalcemia, due to temporary or persistent hypoparathyroidism, for which temporary calcium (Ca) and/or vitamin D supplementation is often needed. Especially for children younger than 3 years, supplementation may be problematic resulting in prolonged length of stay (LOS).^7^ In the debate about the optimal timing of surgery to prevent the development of MTC in MEN2 syndromes, risk and severity of complications should be taken into account. Contrary to studies regarding children 6 years and older, hardly any literature is available on thyroidectomy and its complications in the very young. In this single-center study, we present a unique group of MEN2 children, partly younger than 6 years of age, with long-term follow-up. The aim was to investigate the incidence of postoperative complications after prophylactic thyroidectomy in MEN2 patients, focusing on children younger than 6 years, in an attempt to contribute to the debate of optimal timing of surgery.

## METHODS

### Patients

We conducted a retrospective, single-centered study on children diagnosed with MEN2A and MEN2B undergoing a prophylactic total thyroidectomy at the Wilhelmina Children Hospital (WKZ, currently part of the University Medical Center Utrecht [UMCU]). Only patients aged 17 years or younger when operated on, with surgery after January 1993, were included.

Prophylactic thyroidectomy was defined as surgery on patients with normal preoperative calcitonin levels and no clinically apparent disease. In addition, when levels of basal or stimulated calcitonin were slightly elevated, hence bearing only a moderate risk of MTC and without an anatomical substrate on imaging (ultrasound, computed tomography, magnetic resonance imaging), thyroidectomy was considered prophylactic.^8^ Patients with less than 6 months of serum Ca follow-up were excluded.

This study was reviewed and approved by the institutional review board of the UMCU.

### Laboratory and Genetic Screening

Before 1997, both pentagastrin-stimulated calcitonin levels and basal serum calcitonin levels were measured by radioimmunoassay. After 1997, calcitonin levels were measured by immunoluminometric assay and since April 2006, an immunoradiometric assay (CisBio Bioassays) is used. The appropriate reference levels for each method were used to determine whether the calcitonin levels were normal or elevated.

During the study period, both serum Ca and/or serum ionized Ca (iCa) levels have been measured to evaluate occurrence of hypoparathyroidism after surgery. Serum Ca was measured until 2006 with a Vitros950 (Ortho clinical diagnostics), followed by DxC (UniCel 600 DxC ; Beckman Coulter) until 2012 and since then an AU5811 (Beckman Coulter). Since the introduction in 2003, iCa has been measured by an ion selective electrode on a blood gas analyzer. For both measurements, the laboratory units were consistent from 1993 onwards.

All patients were screened for RET mutation in the National laboratory for Genetics at the UMCU. PCR amplification followed by direct sequence analysis of exons 10–16 of the RET proto-oncogene was used to determine the exact type of mutation. RET mutation was thereafter confirmed on a DNA duplicate sample. The affected codon was used to stratify patients into 3 risk groups according to the 2015 ATA guidelines to define age-appropriate surgery.^4^

### Surgery

Timing of surgery was first guided by our local practice and from 2001 onwards by the leading guideline of that moment.

Every patient underwent a total thyroidectomy by a team consisting of a high volume endocrine surgeon and a high volume pediatric surgeon. From 1993 until 2014, there have been a total of 3 endocrine surgeons and 2 pediatric surgeons at our institution who performed the operation. No routine neck dissections were performed.

### Pathology

The thyroid glands were formalin-fixed and paraffin-embedded according to standard pathology protocols in the UMCU. Regular hematoxylin and eosin stains were performed on all slides and representative slides were subjected to CEA and calcitonin immunostaining. The pathologist evaluated slides for presence of invasiveness, lymph node metastases, and parathyroid glands.

### Postoperative Management

Postoperative hypocalcemia was defined as ≥1 measurements of serum Ca or iCa below reference range, independently of the presence of symptoms. Ca was measured again at least 6 months postoperatively. Hypocalcemia was defined ‘transient’ if serum levels of Ca or iCa had normalized within 6 months. When serum levels were below reference range or if patients were still taking Ca or 1, 25-OH vitamin D medication after 6 months, the hypocalcemia was defined “permanent.”

If serum Ca levels dropped significantly shortly post-operative or did not increase adequately using oral medication (Ca or 1, 25-OH vitamin D), intravenous supplementation was given. When serum levels had normalized or if patients could take their oral supplements at home, they were discharged. For all patients, LOS was registered. Patients with hypocalcemia during their hospital stay including those with additional supplementation at discharge had follow-up serum Ca measurements during outpatient clinic visits until values normalized without treatment.

Damage to the recurrent laryngeal nerve (RLN) was classified transient if there was temporary hoarseness and vocal cord immobility at laryngoscopy. Permanent hoarseness with paralysis of the vocal cord(s) at laryngoscopy was defined as permanent recurrent nerve damage. Postoperative laryngoscopy was not performed systematically in our patients. Vocal cord mobility was investigated using laryngoscopy in patients with postoperative symptoms of possible RLN injury such as hoarseness or stridor. When nerve immobility was observed, further follow-up to classify transient/permanent RLN was done by an ENT specialist.

All patients started thyroid hormone replacement therapy postoperatively. Follow-up was performed regularly by a pediatric endocrinologist. Levothyroxine dose was adjusted depending on thyroid-stimulating hormone and fT4 laboratory results and possible symptoms. Calcitonin levels were measured for the evaluation of recurrent disease.

### Statistical Analysis

In this study, we compared the rate of hypocalcemia and other complications after prophylactic thyroidectomy for children of different ages. Patients were subdivided in very young (<3 year), young (3–6 years), and older (>6 years) children to compare complications in different age groups. Standard descriptive statistics (median, inter quartile range [IQR], range, and frequency) were used to analyze subjects’ characteristics. The analysis of variance was used for comparison of incidence of hypocalcemia, incidence of incidentally removed parathyroid glands and LOS in the 3 different age groups. Student *t* test was used to compare the mean LOS between the group with and without complications. A *P* value <0.05 was considered statistically significant. All data analyses were performed using SPSS version 21.0 (SPSS Inc, Chicago, IL).

## RESULTS

### Baseline

A total of 44 children aged 17 or younger at the time of prophylactic thyroidectomy with at least 6 months of follow-up were included (Table 1). Of these, 18 (41%) were male. The majority of patients had MEN2A syndrome (n = 41, 93%), the remainder having MEN2B syndrome (n = 3, 7%). Based on mutation analysis, 4 patients were classified by the ATA 2015 guideline as highest risk (9%), 37 patient as high risk (84%), and the remaining 3 as moderate risk (7%). The most commonly affected codon was 634 in 37 (84%) of our patients. Mean age at time of surgery was 5.7 years (IQR 3.5–7.9, range 0–17). None of the patients had macroinvasive MTC, lymph node metastases, or distant metastases. Preoperative calcitonin levels were elevated in 20 (46%) patients, 9 of whom had C-cell hyperplasia and 11 microinvasive MTC. A total of 8 patients had a positive pentagastrin-stimulated calcitonin test, all of whom had microinvasive MTC.

**TABLE 1:**
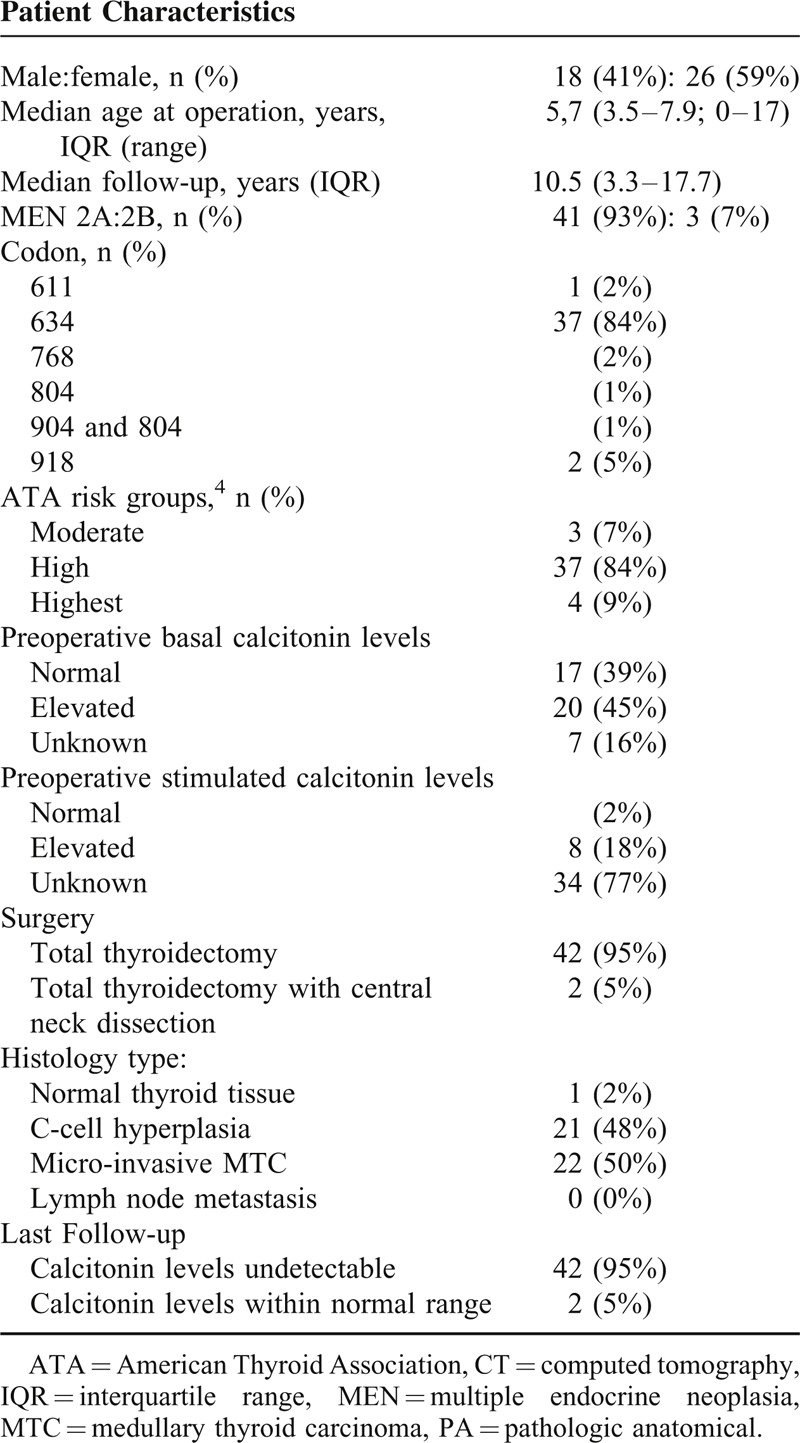
Baseline

Surgery was performed on 9 (20%) patients younger than 3 years (Table 2), 15 (34%) patients 3 to 6 years, and 20 (46%) patients older than 6 years. All patients underwent total thyroidectomy, whereas in 2 patients, an additional central neck dissection was performed because they exceeded the appropriate age for surgery, according to the guideline at that moment. No lymph node metastases were found.

**TABLE 2:**
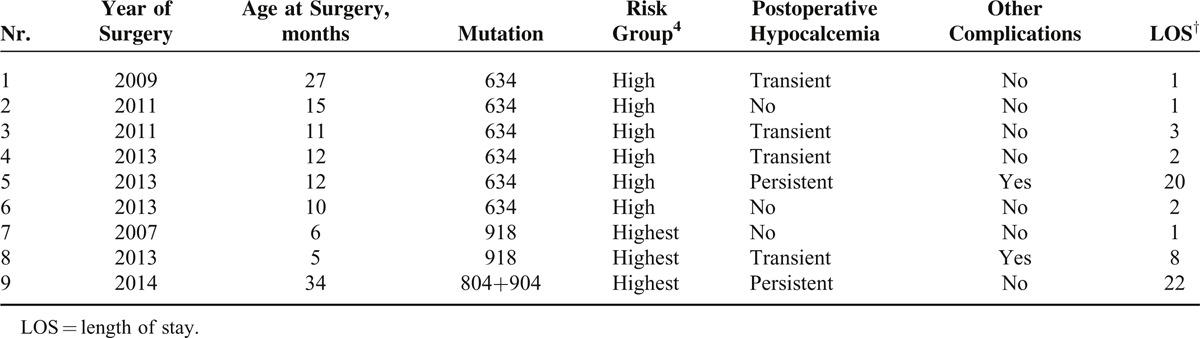
Children With Surgery Younger Than 3 Years

Histological examination revealed normal thyroid tissue in 1 (2%) patient, C-cell hyperplasia in 21 patients, and microinvasive MTC in 22 (50%) patients.

## COMPLICATIONS

### Hypocalcemia

The incidence of transient hypocalcemia was 27% (n = 12). In patients younger than 3 years, 4 (44%) suffered from transient hypocalcemia compared with 5 patients (33%) 3 to 6 years, and 3 patients (15%) older than6 years. Although, in percentage, there was a higher incidence of hypocalcemia with decreasing age, no significant differences were found between the different groups. Only 7 of the 12 patients with transient hypocalcemia required treatment, which consisted of oral or intravenous Ca supplementation and 1, 25-OH vitamin D. Of the 5 patients requiring intravenous supplementation, 3 were younger than 3 years (Table 3). After 6 months of follow-up, these 12 patients were normocalcemic.

**TABLE 3:**
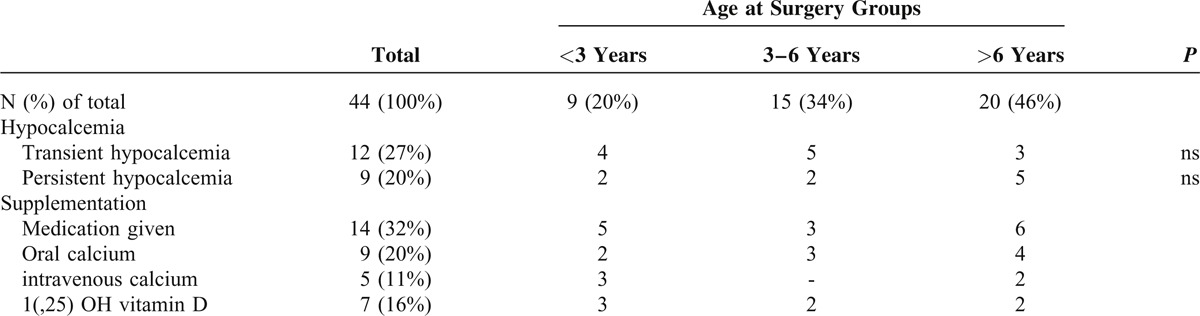
Hypocalcemia in Different Age Groups

Nine (20%) patients were found to have permanent hypocalcemia, requiring oral Ca supplementation and/or 1, 25-OH vitamin D. Permanent hypocalcemia occurred in 2 (22%) patients younger than 3 years, 2 (13%) patients aged 3 to 6 years, and in 5 (25%) patients older than 6 years, including 1 of the 2 patients who underwent additional lymph node dissection. The risk of hypocalcemia did not differ significantly between age groups. All of these patients still received oral supplementation after 6 months of follow-up.

At histological examination, accidentally removed parathyroid glands were found in 9 (20%) patients. All were under 6 years of age at the time of surgery; thus, a significant difference in incidence exists compared with patients older than 6 years (*P* < 0.01). Of the 6 patients in whom 1 gland was found to be removed, 3 were younger than 3 years and 1 had transient hypocalcemia. In 3 patients, 2 parathyroid glands were identified at histological examination of which 2 were younger than 3 years; all developed postoperative hypocalcemia, 2 subsequently showing permanent hypocalcemia.

### Other Complications

Three (7%) patients had other complications, which resulted in a prolonged hospitalization. The first was a 9-year-old child with immediate postoperative stridor caused by transient unilateral recurrent RLN damage, which led to re-exploration of the wound site on the same day; no abnormalities were discovered. Laryngoscopy at follow-up showed complete recovery of vocal cord mobility. The second was a 1-year-old boy who also developed a stridor postoperatively due to bilateral RLN damage requiring re-intubation, dexamethasone, and pediatric intensive care unit (PICU) admission. Dexamethasone in this boy worsened postoperative hypocalcemia. Recovery from thyroidectomy was further complicated by a wound infection requiring both drainage and antibiotics. After 20 days, he was discharged in good clinical condition. Laryngoscopy at 8 months’ follow-up showed permanent unilateral RLN. A 5-month-old boy was postoperatively taken to the PICU for decreased saturation. Following a febrile episode, intravenous antibiotics were given. Furthermore, due to a misplaced intravenous drip containing Ca supplementation, intracutaneous bullae developed, which were treated conservatively.

### LOS

The average LOS for all 44 patients was 3.5 days. For the patients younger than 3 years, this was 6.7 days, versus 1.7 and 3.5 days for the older groups, respectively (*P* < 0.05). For the 22 patients without complications, this was 2.0 days and for the 22 patients with complications 5.0 days (*P* < 0.01).

### Long-Term Outcome

Mean duration of follow-up of all patients was 10.5 years, with a minimum of 4 months and a maximum of 21 years. Forty-two (96%) patients had undetectable calcitonin levels and were considered disease-free at last follow-up. Two (4%) patients had detectable calcitonin levels within reference range; however, no anatomical substrate was found. These patients remain in regular follow-up.

## DISCUSSION

In this study, we investigated the incidence of hypocalcemia and other postoperative complications after prophylactic thyroidectomy in children with MEN2 syndrome. A high rate of hypocalcemia was found in the total patient group when strict definitions are used. Furthermore, thyroidectomy in the very young was associated with a higher overall complication rate and a significantly longer LOS compared with surgery at an older age, whereas none of the patients showed any clinical sign of recurrent MTC.

Over the years, growing evidence has shown a close relationship between type of germline mutation and the rate of progression from C-cell hyperplasia to MTC. This has resulted in a clear shift toward surgery at a younger age, thereby preventing the development of macroinvasive MTC and lymph node metastasis.^9^ However, surgery in young patients has been found to be associated with a higher incidence of complications.^6,10^ In 2014, a consensus statement was published by the European Society of Endocrine Surgeons (ESES), concerning the optimal timing and extent of thyroid surgery.^11^ They conclude that the correlation between surgeon's experience and surgical morbidity is well described, but that the impact of patient's age itself in this context has not yet been clarified.

The largest studies concerning MEN2A/B children mainly focused on the oncological outcome comparing patients of different age groups. Their reported rate of permanent hypocalcemia varied between 2.3% and 7%.^5,12,13,14^ However, these are all multicenter studies, which rarely included patients younger than 6 years and had an unclear registration of complications. Some single-institution series concerning the outcome after thyroidectomy in the normal pediatric population have been reported. However, these studies mainly consist of children undergoing less extensive surgery (eg, unilateral lobectomy for benign thyroid disease) and also hardly if at all include patients younger than 6 years.^15,16^

The largest study that included young children is by Sosa et al who investigated 79 children under 7 years of age. They found that children aged 0 to 6 years had significantly more overall complications (22%), recurrent laryngeal nerve injuries (3.8%), and hypocalcemia (15.7%) compared with older children. In our single-center study including 44 patients of whom 24 were younger than 6 years and 9 younger than 3 years, we found an overall rate of 27% of transient hypocalcemia. This is comparable with the 33% found in a recent study by Kundel et al from the Mayo clinic, albeit much higher than the rate in the general adult population of around 12%.^17,18^ Although not significant, we found a distinct trend toward more hypocalcemia in younger patients with 44% of patients younger than 3 years. Permanent hypocalcemia was found in 9 (20%) patients, which is remarkably higher than the 1.4% to 4% reported in literature concerning pediatric thyroid surgery and the 2% after surgery in adults.^17,19–21^ We found a very high incidence of hypocalcemia in children younger than 3 years, a category rarely included in the other studies. Another reason for the high rate of hypocalcemia could be the difference in definition used. Kundel et al defined permanent hypocalcemia as Ca levels below the normal values while using supplementation. This was the case for 3 (7%) asymptomatic patients in our study, which is comparable with international literature.

In line with previous literature, we found a correlation between the number of parathyroids removed and the incidence of hypocalcemia.^22^ All 3 patients, in whom accidentally 2 parathyroids were removed, developed hypocalcemia, 2 of which were permanent. Moreover, we found that all patients in whom parathyroids were found at histological examination were younger than 6 years. This incidental removal of parathyroid glands differed significantly from the group of patients older than 6 years and is probably because the parathyroids are very small at that age and can be easily missed by the surgeon.

One of the most powerful predictors of poor outcome in pediatric thyroid surgery is having surgery performed by a low-volume surgeon.^6,7^ The observation that individual surgeon experiences is significantly associated with complication rates and LOS after endocrine surgery is also reported for the adult population.^23,24^ All operations in our center were performed by a team consisting of a high-volume endocrine surgeon (>1000 thyroidectomies performed overall) and an experienced pediatric surgeon. Still, two-thirds of the other complications that we described occurred in children younger than 1 year, emphasizing on the difficulty of performing thyroidectomy in this age group.

If complications arise in the very young, they may prove difficult to treat thereby prolonging LOS.^11^ Of the 12 patients with transient hypocalcemia, 5 required intravenous Ca supplementation, 3 of whom were younger than 3 years. In infants, treatment compliance is a problem and safeguarding oral calcium intake might prolong hospitalization. When LOS in children with complications is compared with children without complications, this is significantly different (5.0 vs 2.0 days). This finding matches previous observations wherein children who sustained a complication after thyroidectomy had a significantly longer LOS (4.2 days compared with 1.7 days).^23^

Based on the affected codon, patients are categorized into different risk groups. The most aggressive mutations are represented by the “highest risk” group and there is consensus to operate on these patients as early as possible to prevent development of macro MTC and lymph node metastases. Nonetheless, the most commonly affected codon is codon 634 (84%), categorized as “high risk,” for whom thyroidectomy at the age of 5 is advocated.^4^ This has been a change compared with the 2009 original MTC guideline, that advocated surgery before the age of 5 years.^9^ The guideline, however, does still not provide a minimum age for surgery, therefore allowing surgeons to perform an early thyroidectomy aimed at preventing macroinvasive MTC. We observed in our institution that 6 children of the “high risk” group had surgery before the age of 3 years, of whom 4 developed hypocalcemia (Table 2). If surgery would have been postponed, these complications might not have occurred. Although malignant transformation from C-Cell hyperplasia to MTC might occur as early as at the age of 1 years, the earliest reported case with lymph node metastases in this risk group was aged 5 years and 11 months.^25^ The study by Machens et al^26^ evaluating 130 hereditary MTC cases with codon 634 mutation did not show involvement of regional lymph nodes before the age of 14. Another study from Skinner et al investigating 19 codon 634 mutation patients with a minimum follow-up of 5 years did not show regional metastases before the age of 11 years.^27^ Of the 44 patients we operated, 37 had a mutation in codon 634. Of those 37, none were found to harbor more than micro-MTC, and at long-term follow-up no patient developed clinically recurrent disease. This indicates that we performed surgery at an adequate time and that for some patients there might be room to re-determine the optimal timing of surgery considering complication risks.

Both the 2014 ESES consensus and the revised 2015 ATA guideline suggest that timing of surgery should be guided by serum calcitonin levels, whereby a rise of calcitonin above the normal value marks transition from C-cell hyperplasia to micro-MTC. In our series, however, 9 of 20 patients with elevated calcitonin levels did not show micro-MTC, whereas, on contrary, 11 of 24 patients with normal preoperative calcitonin levels had already developed micro-MTC. Of the 6 high-risk patients younger than 3 years who had surgery, 3 had elevated calcitonin levels but none had developed micro-MTC yet. These findings suggest that we cannot rely solely on the elevation of serum calcitonin in the decision when to perform surgery. In our institution, timing of surgery is based on data regarding transition to micro-MTC for the specific mutation, whereby the wish of parents and serum calcitonin might support decision-making. Of note, we feel calcitonin measurements should be interpreted with caution as in our experience normal calcitonin levels do not preclude micro-MTC. In every case, the risk of developing micro-MTC is carefully measured against the risk of (post-)operative complications.

Our study is, of course, limited by its retrospective design, small patient number, and the long patient inclusion period. However, due to the rarity of MEN2A and 2B, a prospective study with a fair amount of patients will be virtually impossible. Only the ATA high-risk group entails enough patients to make any recommendations on. Our highest- and moderate-risk groups consisted of only 3 and 4 patients, respectively. To draw any conclusions about these groups, a large multi-institutional study is needed. Furthermore, only 2 of our patients had lymph node dissections. Although none of our patients has clinical recurrent disease until so far, we cannot make a statement about the presence of lymph node metastases.

In conclusion, in this large single-center pediatric study with long-term follow-up, we provide evidence that performing thyroidectomy on very young children is associated with a higher rate of hypocalcemia and other complications, causing a significantly increased LOS. Considering this, one should be careful to perform surgery as early as possible in these patients. A balance should be found between the chance of developing macroinvasive MTC and the risk of complications, and timing of surgery should be carefully evaluated. Based on our personal experience and on the available literature, we propose to perform thyroidectomy for the high-risk group not before the age of 3.
